# Randomized clinical trials comparing antibiotic therapy with appendicectomy for uncomplicated acute appendicitis: meta-analysis

**DOI:** 10.1093/bjsopen/zrac100

**Published:** 2022-08-16

**Authors:** Philip J J Herrod, Alex T Kwok, Dileep N Lobo

**Affiliations:** Gastrointestinal Surgery, Nottingham Digestive Diseases Centre and NIHR Nottingham Biomedical Research Centre, Nottingham University Hospitals and University of Nottingham, Queen’s Medical Centre, Nottingham, UK; Gastrointestinal Surgery, Nottingham Digestive Diseases Centre and NIHR Nottingham Biomedical Research Centre, Nottingham University Hospitals and University of Nottingham, Queen’s Medical Centre, Nottingham, UK; Gastrointestinal Surgery, Nottingham Digestive Diseases Centre and NIHR Nottingham Biomedical Research Centre, Nottingham University Hospitals and University of Nottingham, Queen’s Medical Centre, Nottingham, UK; MRC Versus Arthritis Centre for Musculoskeletal Ageing Research, School of Life Sciences, University of Nottingham, Queen’s Medical Centre, Nottingham, UK

## Abstract

**Background:**

This aim of this study was to provide an updated meta-analysis comparing antibiotic therapy with appendicectomy in adults (16 years or older) with uncomplicated acute appendicitis.

**Methods:**

A search for randomized clinical trials comparing antibiotic therapy with appendicectomy in adults with uncomplicated acute appendicitis from inception to 3 October 2021 in MEDLINE, Embase and CENTRAL with no language constraints was performed. Studies were excluded if they included paediatric participants or those with complicated appendicitis. Data on complications of treatment, treatment efficacy (defined in the antibiotic group as not undergoing appendicectomy within 1 year of enrolment, *versus* surgery without complications or no negative histology in the appendicectomy group), readmissions, and length of stay (LOS) were presented.

**Results:**

Eight RCTs involving 3203 participants (1613 antibiotics/1590 appendicectomy; 2041 males/1162 females) were included. There was no significant benefit of antibiotic treatment on complication rates (risk ratio (RR) 0.66, 95 per cent c.i. 0.41 to 1.04). Antibiotics had a reduced treatment efficacy compared with appendicectomy (RR 0.75, 95 per cent c.i. 0.63 to 0.89). Antibiotic treatment at 1 year was successful in 1016 of 1613 (62.9 per cent) participants. There was a six-fold increase in hospital readmissions within 1 year of enrolment in participants receiving antibiotic treatment (RR 6.28, 95 per cent c.i. 2.87 to 13.74). There was no difference in index admission LOS (mean difference 0.15 days (95 per cent c.i. −0.05 to 0.35)).

**Conclusions:**

Earlier optimism regarding the benefits of antibiotic therapy for uncomplicated acute appendicitis does not persist at the same level now that further, large trials have been included. If antibiotic treatment is to be offered routinely as first-line therapy, patients should be counselled appropriately.

## Teaser

Earlier optimism regarding the benefits of antibiotic therapy for uncomplicated acute appendicitis does not persist at the same level now that further, large trials have been included. If antibiotic treatment is to be offered routinely as first-line therapy, patients should be counselled appropriately regarding its shortcomings.

## Introduction

Appendicectomy has been the pillar of management of acute appendicitis^1^ since the description of the classical open procedure by McBurney^2^. Nevertheless, the publication of a case series of successful antibiotic treatment of acute appendicitis^3^ eventually led to the conduct of randomized clinical trials (RCTs) comparing primary antibiotic treatment with appendicectomy for uncomplicated appendicitis^4,5^. Previous meta-analyses by our group^6–8^ of these^4,5^ and subsequent trials^9–11^ demonstrated a reduction in treatment-related complications with antibiotic therapy compared with appendicectomy, without an increase in hospital length of stay (LOS).

Despite these studies, surgery for uncomplicated acute appendicitis remains routine worldwide, with non-operative management being rare. For example, only 31 patients had non-operative management of acute appendicitis in a UK multicentre audit of 5345 patients (0.6 per cent) with acute right iliac fossa pain in 2017^1^ and only 48 in a multinational audit of 4282 patients (1.1 per cent) with acute appendicitis were managed non-operatively in 2016^12^.

Interest in antibiotic treatment for appendicitis was renewed by the challenges of the COVID-19 pandemic when healthcare resources became strained and professional organizations recommended antibiotic treatment as first-line therapy^13^. In the UK, a multicentre observational study reported initial non-operative management in 54 per cent of patients with appendicitis over a 2 month period during the first UK lockdown in 2020^14^.

Since the publication of our last meta-analysis^8^, further large-scale RCTs have been published and we sought to update the evidence from RCTs comparing antibiotic therapy with appendicectomy for uncomplicated appendicitis in adults.

## Methods

This systematic review and meta-analysis was registered prospectively with the PROSPERO database (CRD42021279413, https://www.crd.york.ac.uk/prospero/display_record.php?RecordID=279413) and was performed in accordance with PRISMA Guidelines^15^.

### Search strategy

Literature searches were performed for RCTs on MEDLINE, Embase and Cochrane Central Register of Controlled Trials (CENTRAL) from inception to 3 October 2021 with no language restriction. Search strategies are provided in *Table S1*. Bibliographies of identified potentially relevant studies were hand-searched for further studies. Finally, all studies citing the primary studies on Google Scholar were screened for inclusion.

### Eligibility criteria

Studies were included if they reported an RCT comparing antibiotic treatment with appendicectomy in adult (defined as age 16 years or older) participants with either a clinical or radiological diagnosis of uncomplicated acute appendicitis. Non-randomized studies and studies including patients with complicated appendicitis or paediatric participants were excluded.

### Study selection

Abstracts were screened independently by two authors (P.J.H. and D.N.L.) with the aid of Rayyan systematic review software^16^, with any disagreement being resolved by consensus. Full texts of identified potentially relevant abstracts were then obtained and screened against our selection criteria by two authors (P.J.H. and A.T.K.) with any disagreement resolved by discussion with the senior author (D.N.L.). Where multiple reports describing the same study were identified, data from all reports were used if required, ensuring no double counting of study participants.

### Data extraction

Data were extracted independently by two authors (P.J.H. and A.T.K.), with any disagreement being resolved by consensus. The primary outcome measure of the study was treatment-related complications at 1-year follow-up, defined as any complication of treatment. Secondary endpoints were treatment efficacy at 1 year (defined in the antibiotic group as not undergoing appendicectomy within 1 year of enrolment *versus* surgery without complications or without negative histology (removal of a histologically normal appendix) in the appendicectomy group), readmission to hospital, LOS during index admission, incidence of complicated appendicitis and health-related quality of life (QoL).

### Risk of bias

All included studies were assessed for risk of bias by two authors (P.J.H. and A.T.K.) using the Cochrane Collaboration’s Risk of Bias 2 (RoB 2) tool^17^, with disagreement being resolved by consensus.

### Data synthesis

All data analyses were performed with RevMan 5.4.1 software^18^. For dichotomous variables, risk ratios (RRs) with 95 per cent confidence intervals (c.i.) calculated using a Mantel–Haenszel random-effects model are presented. For continuous variables, mean difference (MD) with 95 per cent c.i. using an inverse-variance random-effects model are presented. All analyses were performed on an intention-to-treat basis. A subgroup analysis was also undertaken, excluding studies that allowed crossover of participants from the antibiotic therapy to the appendicectomy group where appropriate, as in previous iterations of this review^6–8^. Statistical significance was taken at *P* < 0.05 using two-tailed testing. Statistical heterogeneity testing used the *I*^2^ statistic with the following interpretation of values as outlined in the Cochrane Handbook^19^:

- 0–40 per cent, might not be important
- 30–60 per cent, may represent moderate heterogeneity
- 50–90 per cent, may represent substantial heterogeneity
- 75–100 per cent, considerable heterogeneity

Trial sequential analysis^20^ was carried out for the primary outcome of post-treatment complications and the secondary outcome of treatment efficacy using TSA software version 0.9.5.10 (Copenhagen Trial Unit, Centre for Clinical Intervention Research, Copenhagen, Denmark), using a ‘low-biased-based’ estimation of intervention effect, a power of 80 per cent and error of 5 per cent.

## Results

### Search results

**Fig. 1:**
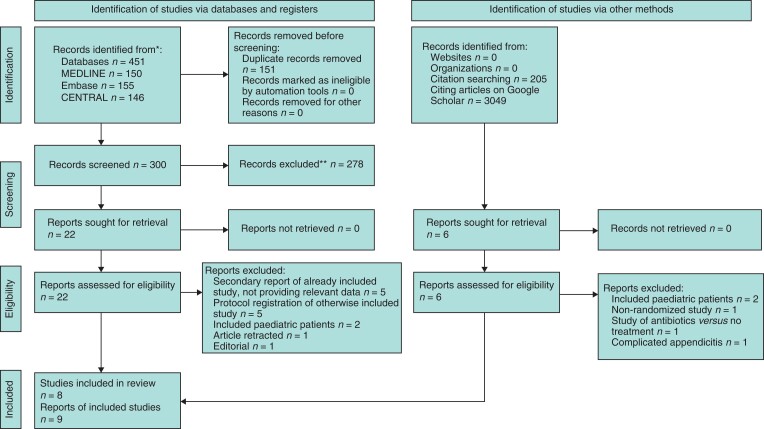
PRISMA flow diagram

*Figure 1* displays the PRISMA flowchart. Eight RCTs met the inclusion criteria^4,5,9–11,21–23^. For one study^22^, data were also extracted from a secondary publication^24^ that provided longer-term follow-up. The characteristics of these studies are described in *Table 1*, and the reasons for exclusion of ineligible studies that had undergone full-text review^25–35^ are listed in *Table S2*.

**Table 1:** **Characteristics of included studies (updated from Rollins *et al***.^8^)

**Eriksson *et al*.^4^**	
Methods	Randomization of participants admitted with history and clinical signs of acute appendicitis.
Participants	Participants with typical history and clinical signs, positive findings at ultrasound and either increased WCC and CRP values or high CRP or WCC on two occasions within a 4 h interval.
Interventions	Antibiotics: cefotaxime 2 g 12-hourly and tinidazole 800 mg for 2 days. Discharged after 2 days with oral ofloxacin 200 mg twice daily and tinidazole 500 mg twice daily for 8 days. Participants were excluded from the study in the event of increased abdominal pain and generalized peritonitis and subjected to surgery. Surgery: underwent open appendicectomy. Treated with antibiotics for 24 h only in the event of bowel perforation or in cases of abdominal spillage. Follow-up: all participants were seen at 6, 10 and 30 days after admission and tested for WCC and CRP, pain scores and temperature recorded. Abdominal and rectal examination was performed on days 6 and 10. Stools were examined for *Clostridium difficile* toxin at day 30. Ultrasound was performed on days 10 and 30.
Outcomes	Pain scores (every 6 h using a visual analogue scale), morphine consumption, WCC and temperature, positive diagnosis at surgery, duration of hospital stay, wound infection and recurrent appendicitis.
**Stryud *et al*.^5^**	
Methods	Participants were asked to participate if appendicectomy was planned, subsequently randomized either to surgery or antibiotic therapy. Participants were monitored at the end of 1 week, 6 weeks and 1 year.
Participants	Men, 18–50 years of age, admitted to six different hospitals between 1996 and 1999. Participants with suspected appendicitis with a CRP concentration above 10 mg/l and with no clinical signs of perforation.
Interventions	Antibiotics: intravenous cefotaxime 2 g 12-hourly and tinidazole 800 mg daily for 2 days. Discharged after 2 days with oral ofloxacin 200 mg twice daily and tinidazole 500 mg twice daily for 10 days. If symptoms not improved within first 24 h, appendicectomy was performed. All conservatively treated participants with a suspected recurrence of appendicitis underwent surgery. Surgery: participants had open or laparoscopic surgery at the surgeon’s discretion.
Outcomes	Duration of hospital stay, sick leave, diagnosis at operation, recurrences and complications.
**Hansson *et al*.^9^**	
Methods	Randomized clinical trial. Three hospitals were included for the study, one hospital used only as a reference cohort for comparison with study and control groups at the other two hospitals. Allocation by date of birth (odd number was antibiotics group and even number was surgery group).
Participants	Participants with positive history, clinical signs, laboratory tests and in some cases, ultrasonography, CT and gynaecological examination.
Interventions	Antibiotics: intravenous cefotaxime 1 g twice daily and metronidazole for at least 24 h. Participants when improved were discharged 24 h later with oral ciprofloxacin 500 mg twice day and metronidazole 400 mg three times a day for 10 days. If no improvement, intravenous treatment was prolonged. Surgery: appendicectomy was performed according to author’s usual practice, single-dose antibiotic prophylaxis, open or laparoscopic technique and postoperative antibiotic treatment when the appendix was gangrenous or perforated.
Outcomes	Treatment efficacy, complications, recurrences and reoperations, duration of antibiotic therapy, abdominal pain after discharge from hospital, duration of hospital stay and sick leave. The total costs for the primary hospital stay were analysed for each patient.
**Vons *et al*.^10^**	
Methods	Open-label, non-inferiority, randomized clinical trial in six academic centres.
Participants	All adults 18 years and older with suspected acute appendicitis. Eligible participants had CT diagnosis of uncomplicated appendicitis, using defined radiological criteria and were randomized to appendicectomy or antibiotic therapy.
Interventions	Participants were assessed twice a day following admission and were discharged after resolution of pain, fever and any digestive symptoms. All participants were seen on days 15, 30, 90, 180 and 360. Antibiotics: intravenous or oral amoxicillin plus clavulanic acid (3 g per day if less than 90 kg or 4 g for participants more than 90 kg) for 48 h. Appendicectomy was performed if there was no resolution of symptoms after 48 h. If symptoms resolved, patients were discharged with antibiotics and reviewed on day 8. CT was performed if persistent pain or fever and possible appendicectomy. If not, antibiotics were continued for another 8 days. If symptoms persisted on day 15, appendicectomy was performed. Surgery: open or laparoscopic appendicectomy according to surgeon’s standard practice. Amoxicillin plus clavulanic acid 2 g at induction of general anaesthesia. Antibiotics were given after surgery only if complicated appendicitis was present.
Outcomes	Primary endpoint: occurrence of peritonitis within 30 days of initial treatment, diagnosed either at appendicectomy or postoperatively by CT. Secondary endpoints: number of days with a post-intervention visual analogue scale pain score of 4 or higher, duration of hospital stay and absence from work, incidence of complications other than peritonitis within 1 year and recurrence of appendicitis after antibiotic treatment (appendicectomy performed between 30 days and 1 year follow-up, with a confirmed diagnosis of appendicitis).
**Salminen *et al*.^11^**	
Methods	Open-label, non-inferiority, randomized clinical trial in six Finnish hospitals. Randomization of participants admitted with CT-proven acute uncomplicated appendicitis between November 2009 and June 2012.
Participants	Participants aged 18–60 years admitted to the emergency department with clinical suspicion of acute uncomplicated appendicitis confirmed by CT were considered. Acute appendicitis was considered present when the appendiceal diameter exceeded 6 mm with wall thickening and at least one of the following: abnormal contrast enhancement of the appendiceal wall, inflammatory oedema, or fluid collections around the appendix. Participants with complicated appendicitis, defined as the presence of an appendicolith, perforation, abscess or suspicion of a tumour on the scan, were excluded.
Interventions	Antibiotics: single daily dose of intravenous ertapenem sodium (1 g/day) for 3 days with first dose administered in emergency department at presentation. Discharged after 72 h of intravenous therapy with 7 days of oral levofloxacin (500 mg once daily) and metronidazole (500 mg three times daily). Participants were excluded from the study if within 12–24 h they went on to develop progressive infection, perforated appendicitis or peritonitis at which point the patient underwent appendicectomy. Surgery: open appendicectomy via McBurney right lower quadrant muscle-splitting approach or laparoscopic appendicectomy. Prophylactic antibiotics (1.5 g cefuroxime and 500 mg metronidazole) administered 30 min before incision. No further antibiotics were administered unless a wound infection was suspected after surgery. Follow-up: all participants seen daily during their hospital stays (days 0, 1 and 2) and subsequently by telephone interviews at 1 week, 2 months and 1 year after the intervention. At 1 week and 2 months pain scores were obtained from a visual analogue scale, sick leave was registered, and the presence of wound infections and recurrent appendicitis was assessed.
Outcomes	The primary outcome measure in the antibiotic group was resolution of acute appendicitis, with discharge from hospital without the requirement for surgical intervention and no recurrent appendicitis during the 1-year follow-up. Treatment success in the appendicectomy group was defined as the patient successfully undergoing an appendicectomy. Secondary outcomes: post-intervention complications, late recurrence of appendicitis (more than 1 year), duration of hospital stay, sick leave taken, pain scores on a visual analogue scale, and the use of analgesics.
**Ceresoli *et al*.^21^**	
Methods	Non-inferiority: Randomized clinical trial at Papa Giovanni XXIII Hospital, Italy, between September 2011 and December 2014. Randomization of all participants fulfilled inclusion criteria within that interval through a computer system.
Participants	Participants aged 18–65 years were diagnosed by AIR score and adjunctive abdominal ultrasound in selected participants. Participants with intermediate probability of acute appendicitis from AIR score were examined with abdominal ultrasound and were included in the study if ultrasound findings confirmed the clinical suspicion of acute appendicitis. Participants with high probability of acute appendicitis from AIR score without signs of perforation and with WCC of less than 15 000/µl and CRP less than 5 mg/l were included for the randomization.
Interventions	Antibiotics: 1 g of ertapenem administered intravenously once a day for 3 days, followed by a further 5 days of co-amoxiclav 1 g use three times per day. Daily AIR score was used during hospitalization to evaluate possible failure. AIR score must be below 5 for discharge. Surgery: preoperative intravenous administration of 1 g ertapenem with appendicectomy in the following 12 h. Laparoscopic appendicectomy with the standard three-port approach was performed routinely. In selected cases, open appendicectomy was indicated based on surgeon’s choice. Only in those with phlegmonous or gangrenous AA seen intraoperatively, ertapenem 1 g once daily was administered for a further 2 days, followed by 5 more days of co-amoxiclav as per the conservative group protocol. Follow-up: all participants were re-evaluated as outpatients after 7 and 14 days from the treatment. Telephone follow-up was conducted after 1 year from the episode of acute appendicitis. In those who were managed conservatively and above 40 years old, a follow-up colonoscopy after 1 month was recommended.
Outcomes	Primary outcome: resolution of symptoms and inflammatory markers (WCC <10 000/µl and CRP <1 mg/l) within 2 weeks after surgery in the surgical group or from the third dose of ertapenem without other treatments in the antibiotic group. Secondary outcomes: complications, negative appendicectomy, duration of hospital stay, work absence, long-term negative outcomes within 1 year, including: bowel occlusion/intraperitoneal abscess leading to surgical re-operation, bowel occlusion longer than 48 h, intraperitoneal abscess, incisional hernia or wound dehiscence in the surgical group and recurrence of acute appendicitis in the antibiotic group.
**CODA trials 2020^22^ and 2021^24^**	
Methods	Non-blinded, non-inferiority randomized trial across 25 US centres with staggering recruitment between 3 May 2016 and 5 February 2020. Randomization was performed by using permuted blocks. Stratification was conducted according to recruitment site and appendicolith status.
Participants	Consecutive English-speaking or Spanish-speaking participants above 18 years of age were approached by the research coordinator if imaging confirmed they had appendicitis. All participants with evidence of appendicolith from imaging results were included in a prespecified subgroup before randomization. Evidence of perforation from the imaging result was not an exclusion criterion.
Interventions	Antibiotics: participants would either be hospitalized for the administration of intravenous antibiotics or received intravenous antibiotics for 24 h/antibiotics with 24 h bioavailability in the emergency departments and discharged without hospitalization. Overall, 47 per cent participants were discharged without hospitalization in the antibiotic group. A 10-day course of antibiotics was given to all participants following the administration of intravenous antibiotics. Some conditions were used as references for proceeding to appendicectomy but the decision to perform appendicectomy was ultimately made by the treating clinician. Surgery: all participants received one dose of antibiotic in the emergency department. Participants may also have received preoperative antibiotics as per local guidance of the centre. The surgical technique for appendicectomy was not standardized with both laparoscopic and conventional approaches allowed but 96 per cent of participants in the group received laparoscopic appendicectomy. Improving clinical criteria, adequate analgesic effects and oral intake of fluids without difficulty were the standard discharge criteria in both groups. Follow-up: participants were followed up by survey at 7, 14 and 30 days and 3, 6, 12, 18, 24, 36 and 48 months from allocation.
Outcomes	Primary outcome: 30-day health status, assessed with EQ-5D™ questionnaires. Secondary outcomes: appendicectomy in the antibiotics group, patient-reported resolution of symptoms, and National Surgical Quality Improvement Program-defined complications at the time of index treatment or during follow-up, visits to the emergency department or hospital related to appendicitis symptoms, appendiceal neoplasms, treatment-related complications, days of missed work for the participants and their career.
**O’Leary *et al*.^23^**	
Methods	Single-centre randomized trial in Ireland aiming to examine the overall efficacy of antibiotic-only treatment of acute uncomplicated appendicitis *versus* surgical intervention. Randomization using online randomization tool. Codes were maintained within a sealed envelope.
Participants	Participants aged 16 years and older admitted to the emergency department with right iliac fossae pain, raised WCC/CRP, fluent in English (and negative β-HCG in females) were screened for inclusion. Participants without exclusion criteria would then proceed to radiological investigation with abdominal ultrasound with/without magnetic resonance imaging performed in those under 45 years; CT in participants above 45 years of age was performed. Participants were randomized if acute uncomplicated appendicitis was evidenced from radiological investigation.
Interventions	Antibiotics: administration of intravenous co-amoxiclav 1.2 g three times daily until clinical improvement was seen, followed by 5 days of 625 mg oral co-amoxiclav three times daily. Surgical: co-amoxiclav 1.2 g administered upon confirmation of diagnosis. A further preoperative dose was administered at the induction of anaesthesia if needed. Three postoperative doses of antibiotics given afterwards. Follow-up: telephone questionnaire and quality of life questionnaire (EQ-5D-3L and EQ-QoL) at 1 week, 1 month, 3 months and 12 months after intervention.
Outcomes	Primary endpoint: success rate of antibiotic treatment at 1-year follow-up for the antibiotic group; successful appendicectomy for the surgical group. Secondary endpoints: quality of life, cost and duration of hospital stay.

β-HCG, human chorionic gonadotropin; AIR, appendicitis inflammatory response; CRP, C-reactive protein; CT, computed tomography; EQ-5D™, European Quality of Life-5 Dimensions; EQ-QoL, EuroQoL quality of life; WCC, white cell count.

### Risk of bias

**Fig. 2:**
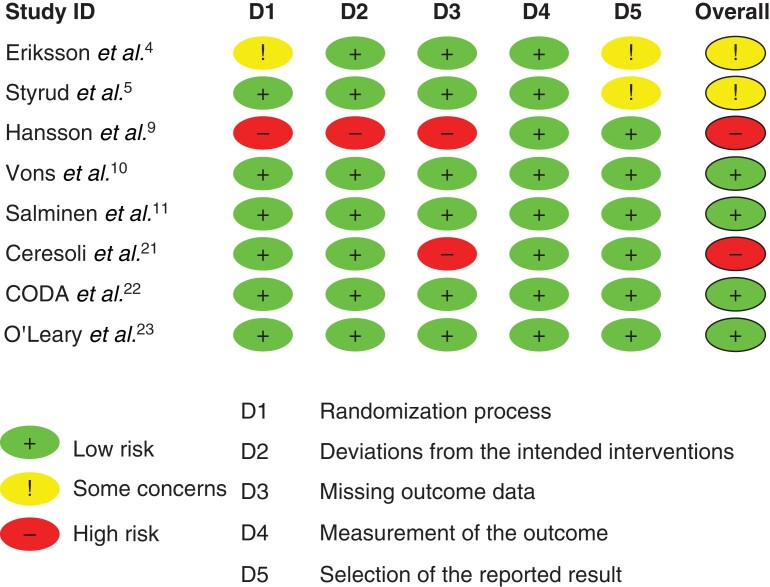
Risk of bias in included studies

*Figure 2* displays the RoB 2 analysis. Four studies^10,11,22,23^ were judged to be at low risk of bias overall. Two studies were judged to have some concerns; one for a lack of detail regarding random sequence allocation^4^, while both studies^4,5^ presented no information to allow a judgement regarding reporting bias. Two studies were judged to be at a high risk of bias; one due to the exclusion of participants found to have complicated appendicitis at operation^21^ and the other for multiple concerns including allowing patient crossover between groups due to patient or surgeon choice, inadequate random sequence generation and missing outcome data^9^.

### Complications

**Fig. 3:**
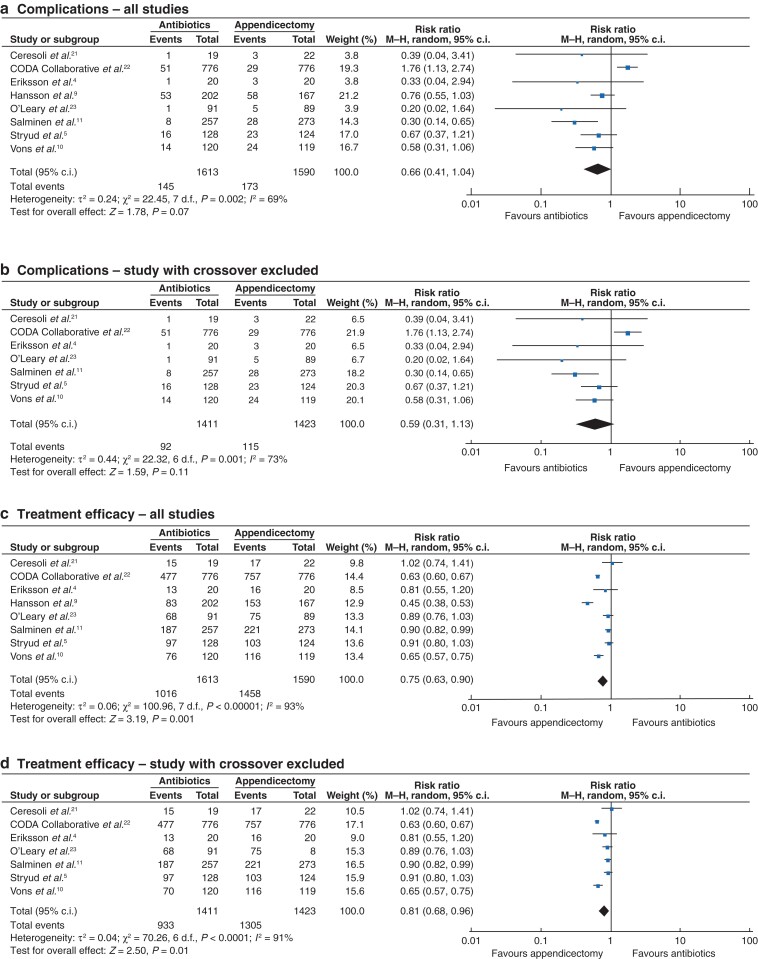
Forest plots comparing complication rates for patients receiving antibiotic therapy *versus* appendicectomy for acute uncomplicated appendicitis **a** all studies and **b** studies with no voluntary crossover of patients and comparing treatment efficacy for **c** all studies and **d** studies with no voluntary crossover of patients. A Mantel–Haenszel random-effects model was used to perform the meta-analyses and risk ratios are quoted including 95 per cent confidence intervals.

All studies reported post-treatment complications. There was no significant difference in the rate of post-treatment complications in participants treated with antibiotics (144 of 1613, 8.9 per cent) compared with those who were randomized to appendicectomy (173 of 1590, 10.9 per cent) (RR 0.66, 95 per cent c.i. 0.41 to 1.04, *P* = 0.07, *I*^2^ = 69 per cent) (*Fig. 3a*). When a subgroup analysis was performed excluding the study that allowed voluntary crossover between groups, there remained no significant difference in post-treatment complications (antibiotics, 92 of 1411, 6.5 per cent *versus* appendicectomy, 115 of 1423, 8.1 per cent) (RR 0.59, 95 per cent c.i. 0.31 to 1.13, *P* = 0.11, *I*^2^ = 73 per cent) (*Fig. 3b*). Trial sequential analysis of this outcome demonstrated the required information size for this outcome to be 6426 patients, which has not yet been reached. The meta-analysis has not yet crossed the boundary for futility (*Fig. S1*).

### Treatment efficacy

All studies reported data to assess treatment efficacy. Antibiotic treatment had a significantly reduced treatment efficacy (1016 of 1613, 63.0 per cent) compared with appendicectomy (1458 of 1590, 91.7 per cent) (RR 0.75, 95 per cent c.i. 0.63 to 0.89, *P* = 0.001, *I*^2^ = 93 per cent) (*Fig. 3c*). When the study that allowed voluntary crossover between groups was excluded, this difference persisted (antibiotics, 933 of 1411, 66.1 per cent *versus* appendicectomy, 1305 of 1423, 91.7 per cent) (RR 0.81, 95 per cent c.i. 0.68 to 0.96, *P* = 0.01, *I*^2^ = 91 per cent) (*Fig. 3d*). Antibiotic treatment was successful at 1 year in 1016 of 1613 (63.0 per cent) participants. Trial sequential analysis of this outcome demonstrated the required information size for this outcome to be 6366 patients, which has not yet been reached, however the Z curve crossed the O’Brien-Fleming boundary, indicating this meta-analysis to be conclusive (*Fig. S2*).

### Length of stay

**Fig. 4:**
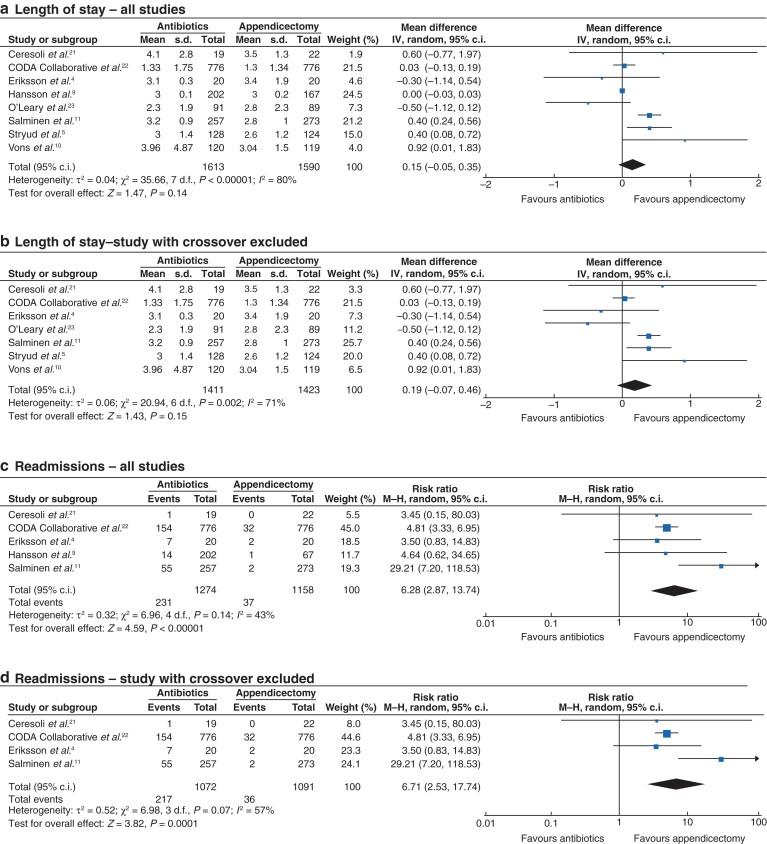
Forest plots comparing index hospital admission length of stay for patients receiving antibiotic therapy *versus* appendicectomy for acute uncomplicated appendicitis **a** all studies and **b** studies with no voluntary crossover of patients. An inverse-variance random-effects model was used to perform the meta-analysis and mean differences are quoted including 95 per cent confidence intervals. Forest plot comparing hospital readmission for patients receiving antibiotic therapy *versus* appendicectomy for acute uncomplicated appendicitis: **c** all studies and **d** studies with no voluntary crossover of patients. A Mantel–Haenszel random-effects model was used to perform the meta-analysis and risk ratios are quoted including 95 per cent confidence intervals.

All studies reported LOS at index hospital admission. There was no significant difference between antibiotic treatment and appendicectomy (MD 0.15 days, 95 per cent c.i. −0.05 to 0.35, *P* = 0.14, *I*^2^ = 80 per cent) (*Fig. 4a*). When the study that allowed voluntary crossover between groups was excluded, there remained no significant difference (MD 0.19 days, 95 per cent c.i. −0.07 to 0.46, *P* = 0.15, *I*^2^ = 71 per cent) (*Fig. 4b*).

### Readmission to hospital

Five studies reported readmission to hospital after completion of index treatment. Participants given antibiotic treatment had a six-fold increase in readmissions to hospital (231 of 1274, 18.1 per cent) compared with those treated with appendicectomy (37 of 1158, 3.2 per cent) (RR 6.28, 95 per cent c.i. 2.87 to 13.74, *P* < 0.001, *I*^2^ = 43 per cent) (*Fig. 4c*). This difference in readmissions remained (antibiotics, 217 of 1072, 20.2 per cent *versus* appendicectomy, 36 of 1091, 3.3 per cent) (RR 6.71, 95 per cent c.i. 2.53 to 17.84, *P* < 0.001, *I*^2^ = 57 per cent) when the study which allowed voluntary crossover between groups was excluded (*Fig. 4d*).

### Complicated appendicitis

Seven studies reported the secondary outcome of complicated appendicitis found either at surgery or on histopathology. The authors of the remaining study^23^ provided this information upon request.

**Fig. 5:**
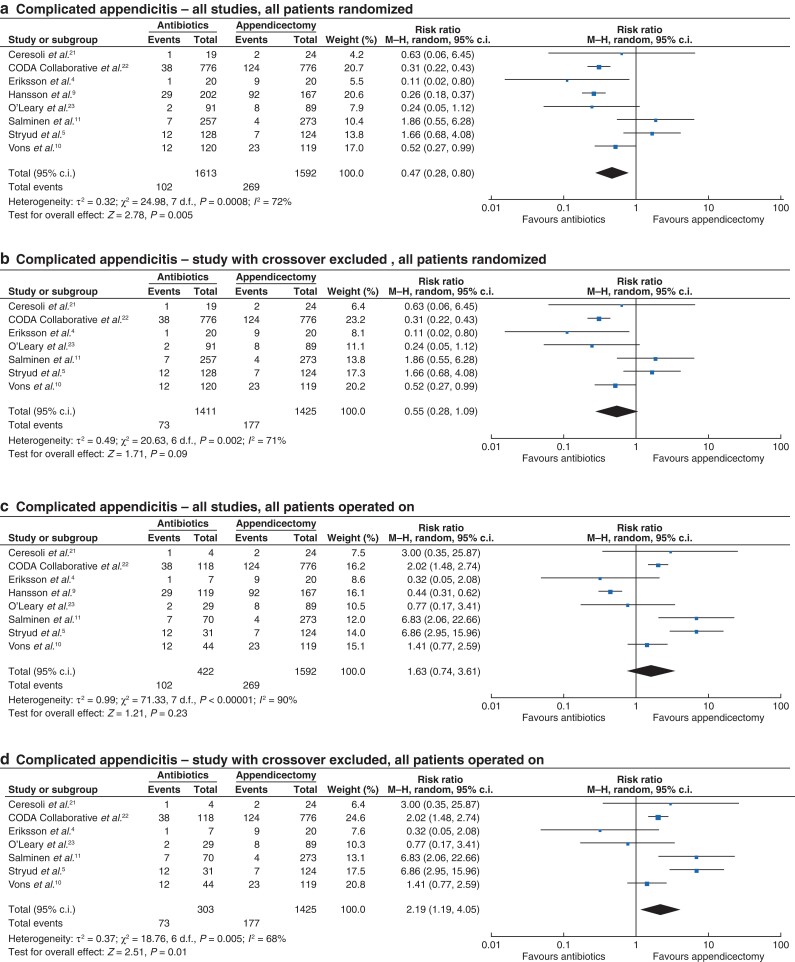
Forest plots comparing incidence of complicated appendicitis found at operation for all randomized patients receiving antibiotic therapy *versus* appendicectomy **a** all studies and **b** studies with no voluntary crossover of patients and incidence of complicated appendicitis found at operation, in only those who underwent an operation for **c** all studies and **d** studies with no voluntary crossover of patients. A Mantel–Haenszel random-effects model was used to perform the meta-analysis and risk ratios are quoted including 95 per cent confidence intervals.

There was a significant reduction in the incidence of complicated appendicitis in all participants allocated to antibiotics (102 of 1613, 6.3 per cent) compared with appendicectomy (269 of 1592, 16.9 per cent) (RR 0.47, 95 per cent c.i. 0.28 to 0.80, *P* = 0.005, *I*^2^ = 72 per cent) (*Fig. 5a*). When the study which allowed voluntary crossover between groups was excluded, this difference was no longer significant (antibiotics, 73 of 1411, 5.2 per cent *versus* appendicectomy, 177 of 1425, 12.4 per cent) (RR 0.55, 95 per cent c.i. 0.28 to 1.09, *P* = 0.09, *I*^2^ = 71 per cent) (*Fig. 5b*).

There was no significant difference in the incidence of complicated appendicitis in participants who failed antibiotic therapy having been initially randomized to this and subsequently proceeding to appendicectomy (102 of 422, 24.2 per cent) compared with those who were randomized to primary appendicectomy (269 of 1592, 16.9 per cent) (RR 1.63, 95 per cent c.i. 0.74 to 3.61, *P* = 0.23, *I*^2^ = 90 per cent) (*Fig. 5c*). When the study that allowed voluntary crossover between groups was excluded, there was a significant increase in the incidence of complicated appendicitis in participants who failed antibiotic therapy and proceeded to appendicectomy compared with those who were randomized to primary appendicectomy (antibiotics, 73 of 303, 24.1 per cent *versus* appendicectomy, 177 of 1425, 12.4 per cent) (RR 2.19, 95 per cent c.i. 1.19 to 4.05, *P* = 0.01, *I*^2^ = 68 per cent) (*Fig. 5d*).

### Quality of life

Two studies reported a QoL metric. The CODA collaborative^22^ reported QoL using the EQ-5D^™^ (EuroQoL Group, Rotterdam, The Netherlands) at a single time point of 30 days after randomization, demonstrating no difference between antibiotic treatment and appendicectomy (mean(s.d.) 0.92(0.13) *versus* 0.91(0.13)).

**Fig. 6:**
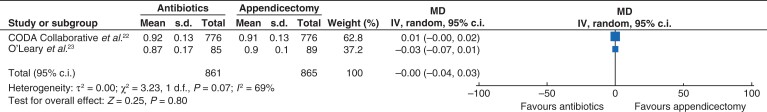
Forest plot comparing quality of life at 30-days for patients in two studies comparing antibiotic therapy with appendicectomy

The COMMA trial^23^ reported QoL using EQ-5D^™^ at four time points (1 week, 1 month, 3 months, and 12 months after randomization); however, data were reported with participants categorized into three groups (appendicectomy, antibiotic treatment, and failed antibiotic treatment with subsequent appendicectomy). The authors reported significantly higher mean (95 per cent c.i.) QoL at 12 months in the appendicectomy group compared with the group that had successful antibiotic therapy (0.976 (0.962 to 0.990) *versus* 0.888 (0.856 to 0.920), *P* < 0.01). Mean (95 per cent c.i.) QoL at 12 months was much lower in the group that had antibiotics with subsequent appendicectomy (0.303 (−0.126 to 0.773)). The study^23^ authors were contacted to provide QoL data according to group allocation. This facilitated meta-analysis with the CODA collaborative study^22^ at the 30-day post-randomization time point, which showed no significant difference in QoL (MD 0.00, 95 per cent c.i. −0.04 to 0.03, *P* = 0.80, *I*^2^ = 69 per cent) (*Fig. 6*).

## Discussion

This meta-analysis of 3203 participants within eight RCTs^4,5,9–11,21–23^ demonstrated that the advantages of primary antibiotic treatment over appendicectomy with regard to post-treatment complications that had previously been shown in earlier meta-analyses are no longer apparent after the inclusion of further large RCTs^21–23^.

The main finding of our group’s 2016 meta-analysis^8^ was that primary antibiotic treatment was associated with fewer post-therapeutic complications; however, in the present meta-analysis this effect is no longer present. There may be several factors contributing to this loss of significance. One may be that the vast majority of appendectomies carried out in the three most recent trials^21–23^ were performed laparoscopically (100 per cent, 96 per cent and 90 per cent), whereas this was much lower in previous studies (the APPAC trial^11^, the largest contributor to our previous meta-analysis, consisted entirely of open procedures). As laparoscopic appendicectomy has been demonstrated to substantially reduce wound infection compared with open appendicectomy^36^, it may be that either the findings of this study are a false negative due to a lower event rate, or that our previous finding of a reduction in complications^8^ was specific to populations in which open appendicectomy is performed more regularly.

Another contributing factor may be that the largest trial^22^ also included participants who were found to have an appendicolith on imaging, whereas some other studies have excluded such participants^11,23^. The presence of an appendicolith is viewed as a risk factor for both perforation and failure of antibiotic therapy in international guidelines^37^ and has been shown on pathological studies to be distinct from appendicitis without an appendicolith^38^. The CODA trial^22^ reported complications in 14 per cent of participants randomized to antibiotic therapy with an appendicolith compared with only 2 per cent of those without an appendicolith. Thus, it may be that this large trial (approximately of a size equal to all the other trials combined) and its inclusion of participants with appendicoliths has affected the analysis significantly, particularly as a secondary analysis of the trial found that presence of an appendicolith was associated with a nearly two-fold increased risk of participants undergoing an appendicectomy within 30 days of initiating antibiotic treatment^39^.

The CODA^22^ and the Vons^10^ trials included participants with an appendicolith on imaging; however, both also routinely imaged their participants with CT before surgery when other trials relied more often on ultrasonography or solely on clinical/laboratory diagnoses. Given that ultrasound has a lower sensitivity for detecting appendicoliths (only 58 per cent in a paediatric population, with the figure likely to be even lower in an adult population)^40^, it may be that some trials may have unknowingly included participants with appendicoliths. Consideration should also be given to the fact that CT is not highly sensitive for complicated appendicitis, with a recent systematic review estimating this to be at 78 per cent^41^. Given that in many countries, cross-sectional imaging is not used routinely in the management of acute right iliac fossa pain in young adults^1^ owing to the risk of exposure to ionizing radiation^42^, it may be that trials such as these may be more relevant to routine clinical practice where the presence of an appendicolith may be unknown.

The other benefits of antibiotic treatment shown in our group’s previous meta-analysis^8^ such as a reduction in LOS have not been demonstrated with the addition of newer trials^21–23^. As with post-treatment complications, this discrepancy may be due to most appendectomies in the more recent studies having been performed laparoscopically, with laparoscopic appendicectomy having previously been demonstrated to result in a shorter LOS than open appendicectomy^36^. In all the studies in this review, antibiotic treatment was initiated intravenously followed by a variable length of oral therapy. However, in the CODA trial^22^ almost half of participants randomized to antibiotics were discharged from the emergency department after receiving intravenous antibiotics with a bioavailability of more than 24 h. Protocols such as this may, in the future, reduce LOS in patients being given primary antibiotic therapy for appendicitis. A reduction in LOS following appendicectomy may, however, also be possible as day-case emergency laparoscopic appendicectomy has been discussed in the literature for more than 20 years^43^.

As in our previous review^8^, antibiotic treatment had a reduced efficacy at 1 year compared with appendicectomy^8^, with a pooled efficacy of 63.0 per cent in the present meta-analysis compared with 91.7 per cent for appendicectomy. This is despite the strict definition of treatment success we chose for appendicectomy, incorporating either negative histology or any postoperative complication as treatment failure. In real-world practice, negative histology rates may be as high as 20 per cent^1^ and resection of a histologically normal appendix is seldom seen as treatment failure. Latest guidelines recommend the removal of the macroscopically normal appendix in symptomatic patients when no alternative pathology is identified at surgery^37^; a view shared in large surveys of surgeons^44–46^. In addition, a recent study has suggested that when resected for right iliac fossa pain, appendices with normal appearances on conventional histology stained positively for pro-inflammatory markers on immunohistochemistry^47^. Thus, it may be that the relative risk of treatment failure with antibiotics is even greater than we report here, given that some of what we have classified as treatment failure for appendicectomy would not be classified as failure in routine clinical practice.

Our meta-analysis has also identified two significant negative consequences of primary antibiotic treatment; an increase in readmission rate and an increase in the risk of complicated appendicitis if appendicectomy is required, findings that were not present in our previous meta-analysis^8^. Antibiotic treatment was associated with a six-fold increase in the risk of readmission to hospital. This is unsurprising, as 37.0 per cent of those randomized to antibiotic therapy required appendicectomy within 1 year, which would have required readmission. It may be that some patients find this an acceptable incidence given the opportunity to avoid index surgery^48^.

The question of antibiotic therapy for acute appendicitis becoming a mainstay of treatment will, however, also rest on the acceptance of patients of this new and non-traditional approach. In a 2018 survey of 1482 patients in America, fewer than 10 per cent of respondents indicated that they would choose primary antibiotic therapy if they were to suffer appendicitis when offered the choice between laparoscopic appendicectomy, open appendicectomy and antibiotic therapy after appropriate counselling^48^. In contrast to this survey, another survey published in research letter form in 2021 showed that 75 per cent of respondents would choose antibiotic therapy even when counselled that the risk of treatment failure could be as high as 60 per cent^49^. Another survey of 245 participants determined that for the treatment of uncomplicated appendicitis, 49.2 per cent preferred antibiotic therapy and 44.5 per cent preferred surgery, whereas 6.3 per cent were undecided^50^. Further high-quality qualitative research will be required to explore the reasons for the starkly contrasting outcomes from these two studies and to understand patient preferences in a variety of circumstances.

A significant limitation of this review was the substantial degree of statistical heterogeneity found in the majority of the meta-analyses that we undertook. This was reduced by performing a subgroup analysis for all outcomes excluding the study by Hansson *et al.*^9^, but remained high. As it was not possible to investigate the effects of radiologically detected appendicoliths on outcomes from primary antibiotic treatment for appendicitis due to the absence of reporting in the trials, a further individual patient data meta-analysis is required to determine this. The risk of bias in the included studies ranged from low in four studies^10,11,22,23^, to high in two^9,21^ with some concerns in the two earliest studies^4,5^. The effect of this on the results of our meta-analyses will have been reduced by the subgroup analyses we performed excluding the study at highest risk of bias.

Trial sequential analysis for post-treatment complications has demonstrated that the required information size for a conclusive meta-analysis is double the number of patients in the current meta-analysis, thus further large trials will be required to conclusively answer the question as to whether antibiotic treatment is associated with fewer complications than appendicectomy; however, this meta-analysis has crossed the O’Brien-Fleming boundary, and thus can be considered conclusive with further trials unlikely to impact on the outcome. If further trials are to be conducted to investigate a benefit to post-treatment complications from primary antibiotic therapy, these must be undertaken with the full knowledge that antibiotic treatment has a significantly reduced efficacy compared with appendicectomy and participants should be counselled regarding this.

This meta-analysis has demonstrated that earlier optimism regarding the benefits of primary antibiotic therapy for uncomplicated acute appendicitis does not persist at the same level after inclusion of newer and larger trials. There seems to no longer be a benefit on post-intervention complications of antibiotic therapy compared with primary appendicectomy and the former carries a significantly increased risk of readmission and an increase in complicated appendicitis in the case of treatment failure or recurrent appendicitis. Primary antibiotic treatment was associated with a treatment efficacy of 63.0 per cent at 1 year, compared with an efficacy of 91.7 per cent for appendicectomy. If primary antibiotic treatment is to be routinely offered as first-line therapy, as during the early stages of the COVID-19 pandemic, it is imperative that patients are counselled appropriately.

## Supplementary Material

zrac100_Supplementary_Data

## Data Availability

All data used in this systematic review and meta-analysis had been previously published in the cited literature, with the exception of additional personal communication from the authors of O’Leary 2021^23^.
