# Supplementary material for: Randomized clinical trials comparing antibiotic therapy with appendicectomy for uncomplicated acute appendicitis: meta-analysis
Source: BJS Open. 2022 Aug 16;6(4):zrac100. doi: 10.1093/bjsopen/zrac100 (PMC9379374; doi:10.1093/bjsopen/zrac100)
Supplement: zrac100_Supplementary_Data [file zrac100_supplementary_data.docx]

**Supplementary material**

**Table S1:** MEDLINE search strategy

| # | Database | Search term | Results |
| --- | --- | --- | --- |
| 1 | Medline | (appendicitis).ti,ab | 20149 |
| 2 | Medline | (appendi*).ti,ab | 41704 |
| 3 | Medline | (appendectomy).ti,ab | 9513 |
| 4 | Medline | (1 OR 2 OR 3) | 45221 |
| 5 | Medline | (antibiotic).ti,ab | 226522 |
| 6 | Medline | (antimicrobial).ti,ab | 172998 |
| 7 | Medline | (antimicro*).ti,ab | 183761 |
| 8 | Medline | (drug therapy).ti,ab | 213246 |
| 9 | Medline | (5 OR 6 OR 7 OR 8) | 578605 |
| 10 | Medline | (4 AND 9) [Document type Randomized Controlled Trial] | 150 |

EMBASE search strategy

| # | Database | Search term | Results |
| --- | --- | --- | --- |
| 1 | EMBASE | (appendicitis).ti,ab | 21991 |
| 2 | EMBASE | (appendi*).ti,ab | 51366 |
| 3 | EMBASE | (appendectomy).ti,ab | 12614 |
| 4 | EMBASE | (1 OR 2 OR 3) | 55987 |
| 5 | EMBASE | (antibiotic).ti,ab | 292107 |
| 6 | EMBASE | (antimicrobial).ti,ab | 227233 |
| 7 | EMBASE | (antimicro*).ti,ab | 242605 |
| 8 | EMBASE | (drug therapy).ti,ab | 51108 |
| 9 | EMBASE | (5 OR 6 OR 7 OR 8) | 536943 |
| 10 | EMBASE | (4 AND 9) [Clinical trials Randomized Controlled Trial] | 155 |

CENTRAL search strategy

ID Search Hits

#1 MeSH descriptor: [Appendicitis] explode all trees 611

#2 MeSH descriptor: [Anti-Bacterial Agents] explode all trees 12590

#3 #1 AND #2 in Trials 146

**Table S2:** Characteristics of excluded studies

| Study | Reference | Reason for exclusion |
| --- | --- | --- |
| Casella, et al. | *Intern Emerg Med* 2011; **6**: 557-8. doi:10.1007/s11739-011-0694-4 | Editorial |
| Haijanen, et al. | *PLoS One* 2019; **14**: e0220202. doi:10.1371/journal.pone.0220202 | Secondary analysis of APPAC trial |
| Khan, et al. | *Med Forum* 2020; **31**: 78-81. | Included paediatric patients |
| Laila, et al. | *Clin Surg* 2017; **2**: 1659. | Included paediatric patients |
| Malik, et al. | *J Gastrointest Surg* 2009; **13**: 966-70. doi:10.1007/s11605-009-0835-5 | Article retracted |
| Mentula, et al. | *Ann Surg* 2015; **262**: 237-42. doi:10.1097/SLA.0000000000001200 | RCT of complicated appendicitis |
| NCT01022567 | <https://clinicaltrials.gov/ct2/show/NCT01022567> | Protocol registration for the APPAC trial |
| NCT01421901 | <https://clinicaltrials.gov/ct2/show/NCT01421901> | Protocol registration for Ceresoli, et al. 2019 |
| NCT02447224 | <https://clinicaltrials.gov/ct2/show/NCT02447224> | Protocol registration for Talan, et al. 2018 |
| NCT02800785 | <https://clinicaltrials.gov/ct2/show/NCT02800785> | Protocol registration for the CODA trial |
| O’Leary, et al. | *Br J Surg* 2020; **107**: 6 doi:10.1002/bjs.12064 | Published abstract of O’Leary, et al. 2021 |
| Paajanen, et al. | *BMC Surg* 2013; **13**: 3. doi:10.1186/1471-2482-13-3 | Protocol registration for the APPAC trial (published) |
| Park, et al. | *Br J Surg* 2017; **104**: 1785-90. doi:10.1002/bjs.10660 | RCT comparing no treatment with antibiotic treatment |
| Salminen, et al. | *JAMA* 2018; **320**: 1259-65. doi:10.1001/jama.2018.13201 | Secondary analysis of APPAC trial |
| Sippola, et al. | *Br J Surg* 2017; **104**: 1355-61.  doi: 10.1002/bjs.10575 | Secondary analysis of APPAC trial |
| Sippola, et al. | *JAMA Surg* 2020; **155**: 283-9. doi:10.1001/jamasurg.2019.6028 | Secondary analysis of APPAC trial |
| Talan, et al. | *Ann Emerg Med* 2017; **70**: 1-11 e9. doi:10.1016/j.annemergmed.2016.08.446 | Included paediatric patients |
| Turhan, et al. | *Ulus Travma Acil Cerrahi Derg* 2009; **15**: 459-62. | Non-randomised study |
| Zaidi, et al. | *Pak Armed Forces Med J* 2017; **67**: 98-101. | Included paediatric patients |

**
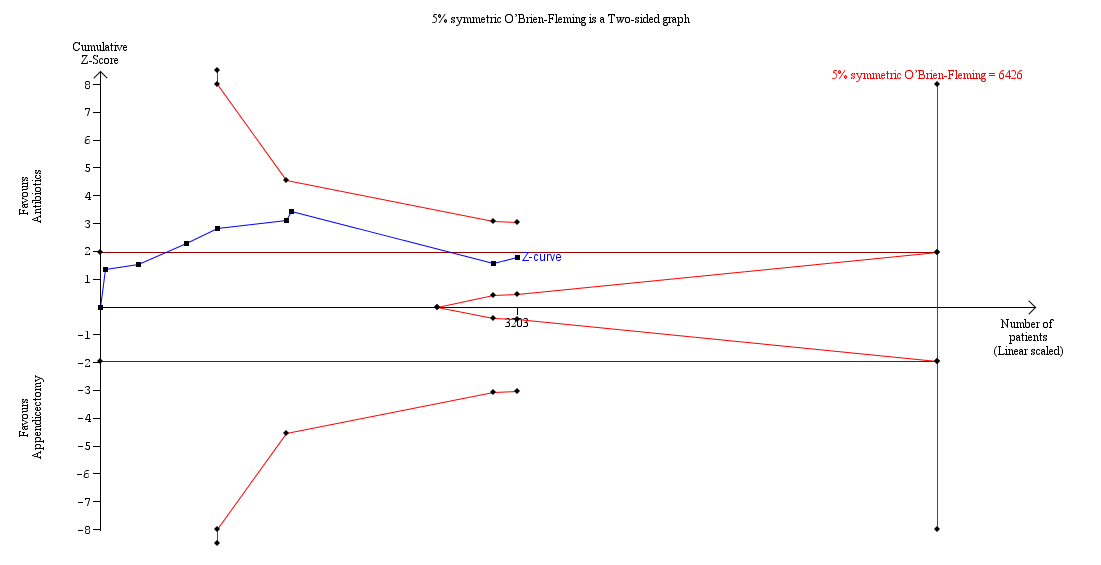
**

**Figure S1:** Trial sequential analysis of primary outcome (post-treatment complications) showing that the required information size has not yet been reached, assuming a power of 80% and an error of 5%.


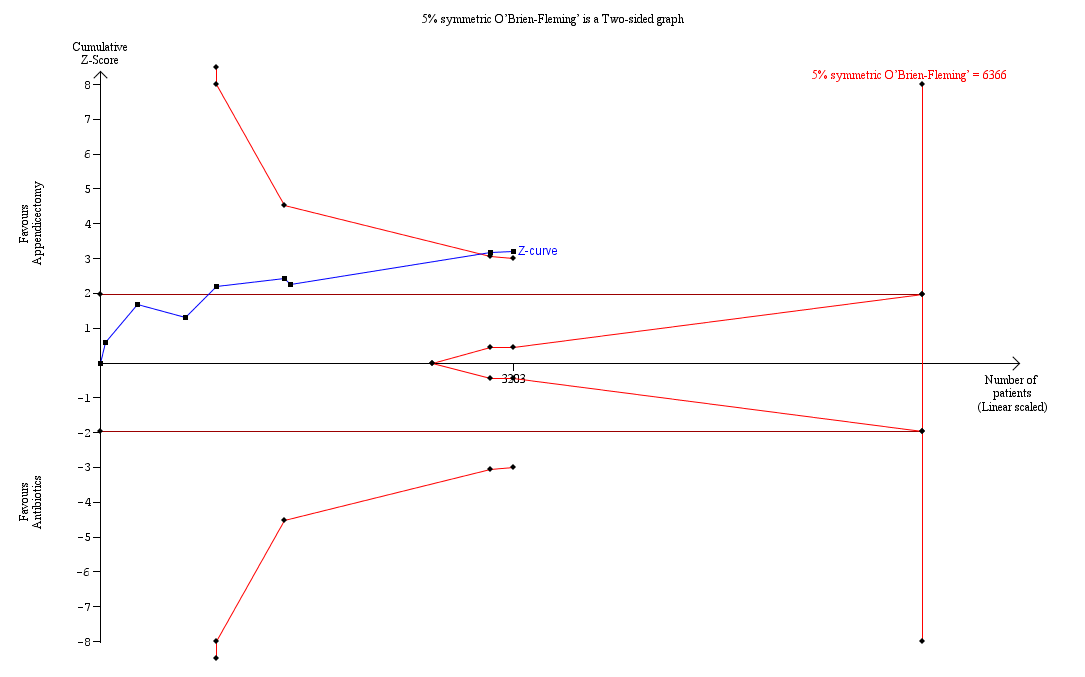


**Figure S2:** Trial sequential analysis of secondary outcome (treatment efficacy) showing that the required information size has not yet been reached however the Z-line crosses the O’Brien-Fleming boundary, assuming a power of 80% and an error of 5%.
